# Nasal Polypi

**Published:** 1908-05-30

**Authors:** 


### Laryngology and Rhinology.

### NASAL POLYPI.

Like many of the older expressions in medical terminology, the word "polypus" has not always borne a precise scientific application: it has, in fact, been applied to any pedunculated tumour springing from a mucous membrane. As concerns the nose, the word should now be restricted to the "mucous polypus," which is a definite pathological entity, although its ætiology is still somewhat obscure.

Nasal polypi, then, are not true neoplasms, but are masses formed by localised œdema of the submucous tissues. They are not composed of œdematous granulation-tissue; nor is there present any myxomatous degeneration, and they have no title to be called myxomata. They present under the microscope the appearance of a loose connective tissue, the fibres of which are widely separated by œdema of the matrix; they are covered by an epithelium which is usually composed of a single layer of ciliated cells, but, if the polypus come down into the inferior meatus and especially if it protrude into the nostril, these may be converted into the squamous variety. Mucous glands are also to be seen, often degenerated and in places dilated to form cysts. The exact mechanism by which these curious growths are formed is obscure, but there is little doubt that they are the result of inflammation and are therefore secondary to some other lesion. The œdema has been variously stated to be due to obstruction of the lymphatics, of the veins, or of the vessels about the ducts of the glands. In at least a large number of cases it can be demonstrated that the bone of the part from which they are growing is the subject of a rarefying osteitis, and isolated spicules of crumbling bone may often be found in the bases of these growths after their removal.

Mucous polypi do not grow from the septum of the nose, nor from the inferior turbinal, floor, or any part of the inferior meatus; they spring most frequently from the ethmoidal region, especially in the middle meatus, but they are also found on the anterior face of the sphenoid or in any of the accessory sinuses of the nose—in fact, on those parts where there is a thin layer of bone lined on each side by muco-periosteum.

They vary in size from minute bodies to enormous masses which fill the cavities of the nose and naso-pharynx, occur singly or in any number, and have usually a narrow pedicle. but also occur as a flattened fringe-like thickening along the border of the middle turbinal body. Typically, they are pale yellowish in colour and translucent, but if they protrude near the nostril or have been inflamed, as after previous operations for removal, they are often pink, firmer, and more opaque. They dimple on probing and are very mobile. Their surface is very shiny and they closely resemble a globule of muco-pus. Though rare in childhood, they occur at all ages of life.

The symptoms they produce are chiefly those of nasal obstruction and discharge; sensations of pressure, headaches, and inability to concentrate the attention are not uncommon. The discharge is a profuse, clear mucus; nasal suppuration is very common in cases of polypus, but the pus comes not from the polypi, but from disease of the accessory sinuses. It has been stated that mucous polypi are always the result of sinus disease. It is possible that, even in cases of single polypus which do not recur after removal there may have been suppuration of a small ethmoidal cell; but the difficult fact to explain is that cases of sinus disease may exist for an indefinite period without producing polypi. Uncomplicated antral empyema is rarely associated with nasal polypi, which are most often found in cases of disease of the ethmoidal labyrinth. Although it is doubtful if sinus-disease is present in all cases of polypi, this is probably always the case where marked suppuration is present as well. Mucous polypi do not cause spontaneous hæmorrhage; polypoid masses which bleed are of a different nature, frequently malignant.

Polypi should be removed with the cold snare. With careful local anæsthesia and skilful manipulation this can be made quite painless. When the growths in front have been removed the anæsthetic should be applied afresh to those which remain. Be careful to wipe away all blood and mucus and to make out accurately the position and relations of each growth before removal, for this is the secret of success. A light snare and flexible wire should be used for the ordinary pedunculated polypi; it is unnecessary to employ for such purpose an instrument powerful enough to cut through the bony turbinal body. The snare must be passed up as near the base of the growth as is possible; the tissue is not cut through, but when firmly constricted is pulled away with a small jerk, for by this means the attachments are removed. One must therefore use a snare with a cross-piece at the distal end of the barrel, and not an instrument in which the wire is completely withdrawn within the tube, for this inevitably cuts through the growth.

Recurrence is to be expected. When polypi have been completely removed, a careful examination is made for suppuration in the accessory sinuses; sometimes on removing a polypus a few drops of pus will suddenly gush out from the site of origin and a probe will be found to enter a diseased ethmoidal cell, which must then be properly opened up. Small buds of polypus and any crumbling bone at the base are to be removed with punch-forceps; the galvano-cautery is worse than useless. Local treatment with sprays and lotions is only indicated when suppuration is present. It is important that the patient should be seen at such intervals as will allow recurrent growths to be removed as soon as they appear, for this offers the best chance of ultimate cure. The slighter cases, with one or two polypi which have grown slowly, are often completely cured at a few sittings, others have to attend once or twice a year for an indefinite time. In the severest cases, after the nose has apparently been completely cleared, when the patient is examined a week or a fortnight later, the polypi are as large and numerous as before, and it is found impossible by removal with the snare to get ahead of their rapid recurrence. For such a general anæsthetic must be given and all the polypi scraped away with a ring-knife and all softened bone removed. The greater part of the ethmoidal labyrinth has frequently to be curetted away and, if necessary, the sphenoidal and maxillary sinuses can be opened up. It need hardly be said that this is a major operation only to be undertaken by those skilled in intra-nasal surgery; recurrences must still be watched for and, if localised, removed with the snare. If this operation be thoroughly performed, the final results are very satisfactory.

